# COVID-19–Induced Modifications in the Tumor Microenvironment: Do They Affect Cancer Reawakening and Metastatic Relapse?

**DOI:** 10.3389/fonc.2020.592891

**Published:** 2020-10-26

**Authors:** Federica Francescangeli, Maria Laura De Angelis, Marta Baiocchi, Rachele Rossi, Mauro Biffoni, Ann Zeuner

**Affiliations:** Department of Oncology and Molecular Medicine, Istituto Superiore di Sanità, Rome, Italy

**Keywords:** coronavirus disease 2019, cancer, dormancy, relapse, inflammation, disseminated tumor cells, tumor microenvironment

## Abstract

Severe coronavirus disease 2019 (COVID-19) causes an uncontrolled activation of the innate immune response, resulting in acute respiratory distress syndrome and systemic inflammation. The effects of COVID-19–induced inflammation on cancer cells and their microenvironment are yet to be elucidated. Here, we formulate the hypothesis that COVID-19–associated inflammation may generate a microenvironment favorable to tumor cell proliferation and particularly to the reawakening of dormant cancer cells (DCCs). DCCs often survive treatment of primary tumors and populate premetastatic niches in the lungs and other organs, retaining the potential for metastatic outgrowth. DCCs reawakening may be promoted by several events associated to severe acute respiratory syndrome coronavirus 2 (SARS-CoV-2) infection, including activation of neutrophils and monocytes/macrophages, lymphopenia and an uncontrolled production of pro-inflammatory cytokines. Among pro-inflammatory factors produced during COVID-19, neutrophil extracellular traps (NETs) released by activated neutrophils have been specifically shown to activate premetastatic cancer cells disseminated in the lungs, suggesting they may be involved in DCCs reawakening in COVID-19 patients. If confirmed by further studies, the links between COVID-19, DCCs reactivation and tumor relapse may support the use of specific anti-inflammatory and anti-metastatic therapies in patients with COVID-19 and an active or previous cancer.

## Introduction

Since the beginning of acute respiratory syndrome coronavirus 2 (SARS-CoV-2) pandemic, several studies have investigated the susceptibility and mortality of COVID-19 in cancer patients. A recent meta-analysis reported a high mortality in patients with COVID-19 and cancer (1), with a worse prognosis in case of progressive cancer, hematological cancer, recent antineoplastic therapies or surgical interventions (2–5). While the impact of cancer and anticancer therapies on COVID-19 mortality is beginning to be understood, several questions concerning the pathophysiology of SARS-CoV-2 infection in cancer patients remain unanswered. Among these, potential long-term effects of COVID-19 on cancer outcome have not yet been explored. In this Perspective, we formulate the hypothesis that severe COVID-19 may increase the risk of subsequent cancer recurrence by inducing the reactivation of dormant cancer cells (DCCs). According to this viewpoint, the major events occurring during severe COVID-19 such as immune-mediated tissue inflammation, impairment of T-cell and natural killer (NK) cell activity, neutrophil hyperactivation and thrombocytosis may collectively generate a temporary pro-tumorigenic microenvironment favorable to DCCs reawakening. Understanding the effects of COVID-19–induced inflammation on tumor cells and their microenvironment will be crucial for a thorough evaluation of the potential long-term risks of COVID-19 in cancer patients and for the implementation of anti-inflammatory and anti-metastatic therapeutic schedules.

## Inflammation, Metastatic Reawakening, and Cancer Recurrence

Cancer recurrence can occur after the apparently successful treatment of solid tumors, due to the presence of residual neoplastic cells in the primary tumor area or at metastatic sites. Metastatic recurrence is responsible for over 90% of cancer deaths and depends on the ability of tumor cells to migrate, seed other organs and restart to proliferate, often after an asymptomatic period named metastatic dormancy (6). During this period, pre-metastatic cells implement an array of strategies to ensure their survival and escape from immune surveillance (7). Among such strategies, adopting a non-proliferative state allows DCCs to persist for long time at metastatic sites, giving rise to tumor recurrence years or even decades after diagnosis. The events responsible for DCCs reactivation are only partly understood. Although it cannot be excluded that cell-intrinsic signals such as additional oncogenic mutations may cause DCCs reawakening, several studies have linked metastatic recurrence to microenvironmental cues such as inflammatory or immune-mediated signals (8). Metastatic reawakening has been reported to be triggered by disruption of tissue homeostasis that usually occurs during acute or chronic inflammation (8, 9). Inflammation has been linked to metastatic recurrence in a variety of conditions including obesity (10) or surgical removal of primary tumors (11). Also pathogen-induced infection has been reported to promote the migration of cancer cells to metastatic sites (12) and the reactivation of dormant metastatic cells (13–15). In addition to metastatic reawakening, acute infection by respiratory viruses has been shown to induce an exhaustion of CD8^+^ T cells that may contribute to release pre-metastatic cells from immune-mediated control (16). Altogether, these evidences indicate that inflammation plays an important role in dictating cancer recurrence and suggest that a defined pro-inflammatory event such as pathogen-induced inflammation seems sufficient to promote DCCs reawakening and metastasis (13–15).

## Role of ACE2 in COVID-19 and in the Regulation of Inflammatory Pathways

**Figure 1:**
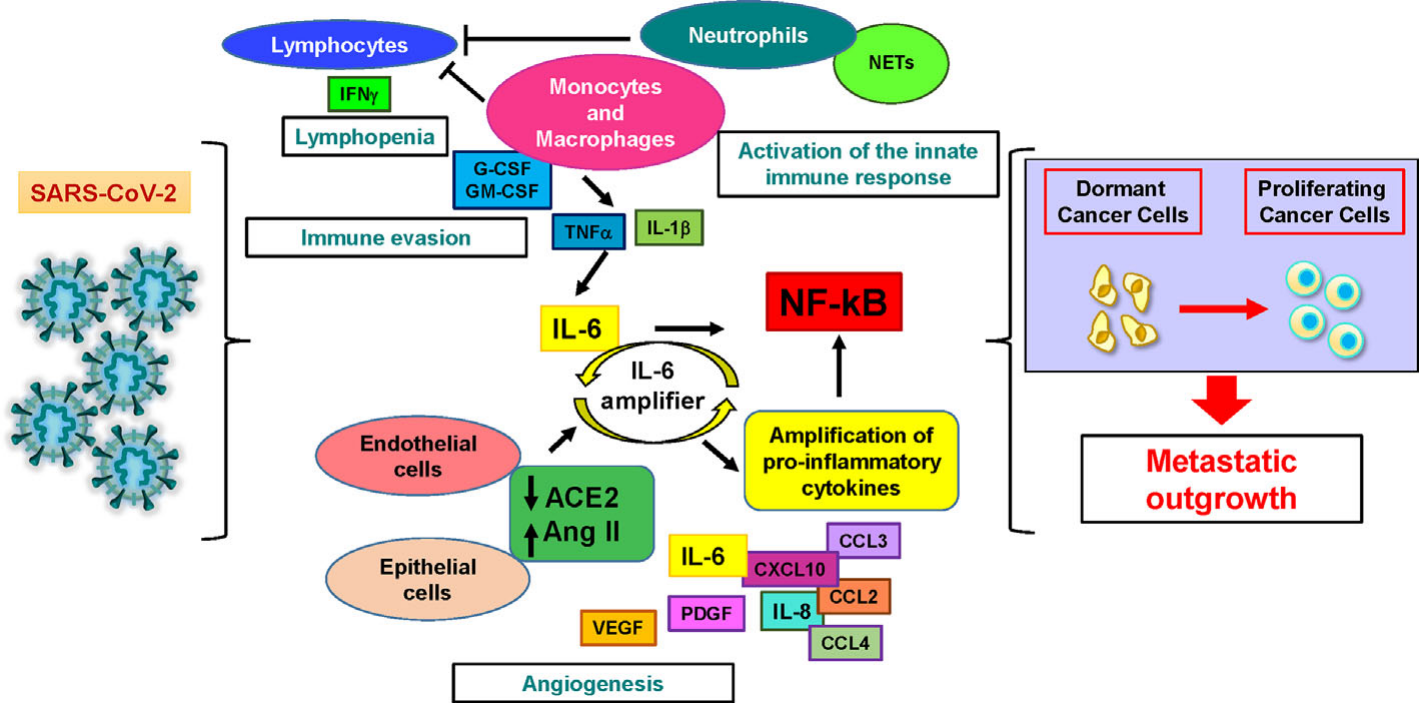
SARS-CoV-2 infection may induce dormant cancer cell proliferation and metastatic relapse. Cellular and molecular factors involved in the pathogenesis of severe COVID-19 play also multiple roles in cancer. Lymphocytes are activated during the first phase of the disease and produce interferon-gamma (IFNγ), then their numbers and activity decrease, resulting in lymphopenia. Activated innate immune response cells (neutrophils and monocytes/macrophages) sustain immune evasion by depressing lymphocyte activity and hindering lymphocyte access to the tumor. They also trigger the production of interleukin-6 (IL-6), starting the systemic release of proinflammatory cytokines and chemoattractants by immune and non-immune cells. Interleukin-1β (IL-1β) and tumor necrosis factor α (TNF-α) further stimulate the production of IL-6. In virus-infected epithelial and endothelial cells, the downregulation of angiotensin-converting enzyme-2 (ACE2) that follows SARS-CoV-2 entry releases the brake from angiotensin II. This event stimulates additional IL-6 production by activating the IL-6 amplifier, a positive feedback loop leading to the uncontrolled production of pro-inflammatory factors. At the same time, neutrophil extracellular traps (NETs) generated by activated neutrophils physically obstruct the access of lymphocytes to inflamed tissues and promote the reawakening of dormant cancer cells. Additional cytokines increased during COVID-19 include granulocyte colony-stimulating factor (G-CSF), granulocyte-macrophage colony-stimulating factor (GM-CSF) (which stimulate neutrophil and monocyte expansion), platelet derived growth factor (PDGF) and vascular endothelial growth factor (VEGF) (which may contribute to tumor angiogenesis). All these events may generate a microenvironment favorable to the proliferation of dormant tumor cells and to subsequent metastatic outgrowth.

Shortly after the beginning of the COVID-19 pandemic, ACE2 was identified as the entry receptor for SARS-CoV-2 and the serine protease TMPRSS2 as the responsible for spike (S) protein priming (17). Following entry, the S protein is cleaved by endosomal acid proteases, thereby releasing the viral genome. Subsequent steps of viral replication, assembly and release have been described in detail for SARS-CoV (18). ACE2 downregulation upon viral infection triggers a cascade of events that contribute to the catastrophic consequences of severe COVID-19 (19). Since ACE2 has been reported to exert multiple anti-tumor effects including inhibition of cancer angiogenesis and metastasis, its downregulation may *per se* promote tumor progression (20–22). Second, ACE2 is responsible for the conversion of angiotensin II (AngII) to angiotensin 1–7, a process that plays an important role in the control of inflammation and cardiovascular homeostasis by the renin-angiotensin system (RAS) (23). An alteration in the respective levels of AngII/Ang(1–7) can result in vasoconstrictive, proinflammatory, and prothrombotic effects, possibly contributing to the renal and cardiovascular complications observed in COVID-19 patients (24). RAS imbalance that follows SARS-CoV-2 infection has been also proposed to be responsible for an increased expression of TGF-β and pro-inflammatory cytokines that collectively promote lung fibrosis (25). Importantly, the AngII/AT1R axis acts on a variety of non-immune cells to activate nuclear factor-κB (NF-κB), a transcription factor essential for inflammatory responses (26). Moreover, AngII stimulates the release of soluble IL-6 and the subsequent activation of STAT3 (27), contributing to activate the IL-6 amplifier (**Figure 1**). NF-κB hyperactivation consequent to ACE2 downregulation cumulates with NF-kB activation induced by MyD88 and pattern recognition receptors activated by viral particles, thus becoming a central molecular event in coronavirus clinical picture (28). NF-κB is the most important molecule linking inflammation to cancer. NF-κB activation in cancer cells promotes proliferation, chemoresistance, epithelial-to-mesenchymal transition, stemness and invasion, while in the tumor microenvironment (TME) it stimulates angiogenesis and immune suppression, collectively supporting the metastatic process (29).

## IL-6 and IL-1 Signaling in COVID-19 and Cancer

COVID-19 patients admitted to intensive care units usually develop acute respiratory distress and cytokine release syndrome (CRS), a life-threatening toxicity that may lead to sustained fever, edema, neurologic symptoms, organ failure and shock (26). Cytokines found to be elevated in the plasma of patients with severe COVID-19 include interleukin-1β (IL-1 β), IL-6, IL-7, IL-8, IL-9, IL-10, granulocyte colony stimulating factor (G-CSF), granulocyte-macrophage colony-stimulating factor (GM-CSF), interferon γ (IFN-γ), macrophage inﬂammatory proteins (MIP)-1α and β (also called CCL3 and CCL4), monocyte chemoattractant protein-1 (CCL2), C-X-C motif chemokine 10 (CXCL10), platelet derived growth factor (PDGF), tumor necrosis factor α (TNF-α), and vascular endothelial growth factor (VEGF) (30–32). IL-6 plays a central role in the pathophysiology of CRS. Accordingly, it was found to be elevated in hospitalized COVID-19 patients and associated with more severe form of the disease (30, 33). Uncontrolled IL-6 signaling may result in inflammation and tissue damage (34). During SARS-CoV-2 infection IL-6 signaling is activated in both immune and non-immune cells triggering the so-called IL-6 inflammation amplifier, a positive feedback loop resulting in the production of additional IL-6, of VEGF and of multiple chemoattractant proteins (35) (**Figure 1**). In parallel to its key role in immune-mediated inflammation, IL-6 sustains tumorigenesis both through a direct stimulation of cancer cells and through an indirect action on the TME. IL-6 stimulates cancer growth, metastasis and immune evasion in several tumor types (36–42). Additionally, direct effects of IL-6 on cancer cells include an ability to promote stem cell characteristics (42–46), induction of mesenchymal traits (39) and resistance to therapies (47). IL-6 orchestrates the interactions between cancer cells and the TME by preparing the soil for cancer homing at target organs (36, 48) and has been also shown to stimulate immune evasion by inducing the stabilization of programmed death-ligand 1 (PD-L1) (38). A mechanistic link between IL-6 and DCCs reactivation is still missing, but elevated levels of IL-6 have been correlated with increased rates of tumor relapse in breast cancer and head and neck cancer (49, 50) while inhibition of IL-6/STAT3 signaling reduced cancer recurrence in preclinical models of breast, head and neck and hepatocellular carcinoma (51–54). IL-6 signaling is characterized by an extreme complexity, which derives at least in part by the promiscuous utilization of signaling components by multiple family members (55). In addition to its pro-tumorigenic effects, IL-6 has also been shown to exert anti-tumor effects by increasing T cell trafficking and adhesiveness to the tumor endothelium (56). Altogether, IL-6 may play multiple and possibly contrasting roles in patients with cancer and COVID-19, which will need to be addressed by future studies. Besides IL-6, also IL-1β has been reported to be elevated in COVID-19 patients as compared to controls (30). High doses of the recombinant IL-1R antagonist anakinra provided clinical improvement in COVID-19 patients (57). Similar to IL-6, also IL-1β has been reported to play a complex role in inflammation and cancer. The pro-tumorigenic role of IL-1β seems to be prevalent, as this cytokine drives chronic inflammation, recruits myeloid-derived suppressor cells (MDSCs), enhances neoangiogenesis and promotes invasion and metastasis (58). However, some anti-tumor effects of IL-1β have been reported (59). A mechanistic insight between IL-1β, inflammation and cancer was recently provided by Wellenstein and coworkers by showing that IL-1β is responsible for neutrophil expansion and neutrophilic inflammation that potentiates the metastatic progression of breast cancer (60). Future investigations will be essential to clarify whether IL-6, IL-1β, and other pro-inflammatory cytokines produced during COVID-19 may affect tumor cells and the TME, possibly supporting the use of anti-cytokine therapies in cancer patients with COVID-19.

## Neutrophils and Neutrophil Extracellular Traps: Double Players in COVID-19 and Tumor Reawakening

Among cells of the innate immune system, neutrophils play a prominent role in fighting microbial infections but also in inflicting tissue damage. Furthermore, neutrophils are involved in a network of inflammatory reactions that promote all the stages of tumor initiation, progression, angiogenesis and metastasis (61, 62). Neutrophils generate reactive oxygen and nitrogen species, release proteases, arginase, ectonucleotidases, matrix metalloproteinases, prostaglandin E2, cyclooxygenases, IL-10 and TGFβ1 (62) and express Fas-ligand and PD-L1, which induce lymphocyte apoptosis and immune suppression (63, 64). Once they reach the tumor microenvironment, neutrophils may undergo transition to MDSCs that inhibit CD4^+^ and CD8^+^ tumor-inﬁltrating lymphocytes, as well as stimulating tumor growth, angiogenesis and metastasis (61). Besides releasing soluble pro-inflammatory factors, activated neutrophils produce Neutrophil Extracellular Traps (NETs), stretches of DNA and globular protein domains that aggregate into large 3D structures (65). NETs provide for a high local concentration of antimicrobial components and for a physical barrier preventing further pathogens spreading. However, NETs can also have a deleterious effect on host tissues, being involved in the pathogenesis of infectious, inflammatory and thrombotic disorders. An early study reported an intense neutrophilic infiltration of pulmonary interstitial spaces and alveoli, and proposed a role for NETs in COVID-19 (65). Lately, NET components have been detected in the sera of COVID-19 patients, being higher in cases requiring mechanical ventilation (66). Subsequently, the presence of NETs and of NET-specific marker myeloperoxidase/DNA complexes in COVID-19 patients was reported by other investigators (67–69) and was related to immunothrombosis (67). NETs have been also shown to play multiple roles in cancer. NETs contribute to tumor immune evasion by coating tumor cells and protecting them from CD8^+^ T cell- and NK-mediated cytotoxicity (70). Moreover, NETs have been reported to promote metastasis formation through several mechanisms including the capture of circulating tumor cells (71), the activation of pro-metastatic fibroblasts (72) and the proteolytic destruction of the antitumorigenic factor thrombospondin-1 (15). Importantly, NETs have been reported to awaken dormant breast cancer cells disseminated in the lungs. In fact, laminin destruction by NET-associated proteases can activate integrin signaling in lung-resident DCCs, triggering integrin-mediated activation of focal-adhesion kinase and ultimately resulting in tumor cell reactivation (13). In summary, neutrophils and their antimicrobial products may contribute not only to COVID-19-associated inflammation and immunothrombosis but also to the reawakening of DCCs disseminated in the lungs and possibly in other organs.

## Potential Roles of Impaired T-Cell Responses in COVID-19 and DCCs Reawakening

Lymphopenia with drastically reduced numbers of circulating T cells (particularly striking for CD8^+^ T cells in patients requiring intensive care) and a functional impairment of NK cells have been consistently detected in severe COVID-19 cases (73). COVID-19–associated lymphopenia can be more severe and persistent as compared with other viral infections and seems to be more selective for T cell lineages (74). Also, it appears to impact prevalently CD8^+^ T cells, although also CD4^+^ T cells are affected (74). It is possible that the peripheral lymphopenia observed in COVID-19 patients reflects the recruitment of lymphocytes to the inflamed lungs. However, autopsy studies and single-cell RNA sequencing of bronchoalveolar lavage fluid did not highlight an excessive lymphocytic infiltration, suggesting that pulmonary sequestration of lymphocytes is not the main cause of lymphopenia in COVID-19 patients. More likely, the causes of lymphopenia during COVID-19 are multifactorial, possibly include extensive lymphocyte death, inhibition by the inflammatory cytokine milieu and indirect effects exerted by other cell types such as dendritic cells and neutrophils (73, 74). The impact of COVID-19–associated T cell and NK cell alterations on tumor cells is still unknown and will likely depend by context-specific and tumor-specific factors. However, both CD8^+^ T cells and NK cells have a crucial function in immune-mediated dormancy and their depletion has been shown to release the brakes from DCCs leading to metastatic outgrowth (75, 76). Latent cancer cells were also shown to persist long time by evading NK-mediated immune surveillance through downregulation of cell surface innate immune sensors (77). Therefore, lymphopenia may contribute, together with inflammation-related factors, to create a microenvironment favorable to metastatic reawakening.

## Therapeutic Strategies for COVID-19 That Interfere With Pro-Tumorigenic Pathways

Hyper-inflammation crucially contributes to COVID-19 severity and patient death, and dexamethasone is the first drug shown to improve patient survival (78). Multiple anti-inflammatory agents are thus currently undergoing clinical evaluation for COVID-19, including not only corticosteroids but also biologicals that target inflammatory cytokines, such as anti-IL-6 or anti-IL-1β agents, and other immune-modulatory agents (**Table 1**). Drugs that block IL-1β signaling may also inhibit the NET-IL-1β loop and decrease NETs formation (65). Additional drugs that interfere with NETs (although not specifically) are inhibitors of neutrophil elastase, recombinant DNases and colchicine (65). Trials evaluating the blockade of additional myeloid-derived inflammatory cytokines, such as TNF57 and granulocyte–macrophage colony- stimulating factor (GM–CSF) are also being considered and/or initiated (79). Other strategies to reduce hyperinflammation in patients include targeting common downstream mediators of cytokine signaling, such as JAK proteins (downstream of IL-6 receptor and several other cytokine receptors) (80, 81) or IRAK4 (that mediates Toll-like receptor (TLR) and IL-1β signaling). The Bruton’s tyrosine kinase inhibitor acalabrutinib, used for chronic lymphocytic leukemia (CLL) and involved in the inhibition of TLR7/8, has shown beneficial effects on CLL/COVID-19 patients (82) and is currently undergoing clinical trials for COVID-19. Drugs against chemokine receptors may reduce CRS in COVID-19 patients by inhibiting massive monocyte infiltration in the lungs or other organs. Accordingly, trials with anti-CCR5 (leronlimab) and anti CCR-2 (cenicriviroc) antibodies have been initiated in patients with COVID-19. Modulating the interferon response may also be useful in reducing inflammation during COVID-19. Clinical trials have been initiated testing the administration of type I interferons (IFNαβ) or type III interferon (IFNλ), which are potent activators of the antiviral response. By contrast, IFNγ production likely contributes to macrophage hyperactivation and tissue damage. Therefore, trials to evaluate IFNγ blockade with emapalumab are underway. In addition to short-term therapies aimed at reducing the deleterious consequences of COVID-19–associated hyperinflammation, long-term approaches to reduce the risks of inflammation-related metastasis may be evaluated by future clinical studies in cancer patients with COVID-19. Targeted approaches against critical receptors for metastatic niche components (such as integrin α_v_β_3_) have been proposed as a feasible strategy to prevent DCCs reactivation (83). By contrast, long-term treatment of cancer patients with anti-inflammatory drugs should be carefully evaluated, as corticosteroids have been reported to increase breast cancer metastasis (84) while non-steroidal anti-inflammatory drugs increase the risk of venous thromboembolism (85). In all cases, strict adherence to recurrence monitoring schedules may be recommended for patients with COVID-19 and an ongoing or previous history of cancer.

**Table 1:** Possible therapeutic targets linked to inflammation and cancer in COVID-19.

Pathway/molecular target	Potential role in COVID-19	Drug type	Drug name	Clinical trials for COVID-19 (clinicaltrials.gov)
Inflammatory cells and mediators	Pro-inflammatory	corticosteroid	dexamethasone	31 studies https://clinicaltrials.gov/ct2/results?cond=COVID-19&term=Dexamethasone&cntry=&state=&city=&dist=&Search=Search
			hydrocortisone	11 studies https://clinicaltrials.gov/ct2/results?cond=COVID-19&term=Hydrocortisone&cntry=&state=&city=&dist=&Search=Search
IL-6 signaling	Pro-inflammatory	anti-IL-6 receptor	tocilizumab	63 studies https://clinicaltrials.gov/ct2/results?cond=COVID-19&term=Tocilizumab&cntry=&state=&city=&dist=&Search=Search
			sarilumab	16 studies https://clinicaltrials.gov/ct2/results?cond=COVID-19&term=Sarilumab&cntry=&state=&city=&dist=&Search=Search
		anti-IL-6	siltuximab	4 studies https://clinicaltrials.gov/ct2/results?cond=covid-19&term=Siltuximab&cntry=&state=&city=&dist=
			clazakizumab	6 studies https://clinicaltrials.gov/ct2/results?cond=COVID-19&term=Clazakizumab&cntry=&state=&city=&dist=&Search=Search
IL-1β signaling	Pro-inflammatory, NET-IL-1β loop	IL-1R antagonist	anakinra	26 studies https://clinicaltrials.gov/ct2/results?cond=COVID-19&term=Anakinra&cntry=&state=&city=&dist=&Search=Search
	Pro-inflammatory	Anti-IL-1β	canakinumab	6 studies https://clinicaltrials.gov/ct2/results?cond=Covid-19&term=Canakinumab&cntry=&state=&city=&dist=
NETs formation	Pro-inflammatory, immune suppression	Neutrophil elastase inhibitors	alvelestat	NCT04396067
			lonodelestat, elafin	N/A
NETs structural integrity	Pro-inflammatory, immune suppression	Recombinant DNAses	dornase	8 studies https://clinicaltrials.gov/ct2/results?cond=COVID-19&term=Dornase&cntry=&state=&city=&dist=&Search=Search
			alidornase alpha, dnase 1 like 3	N/A
IFNγ	Pro-inflammatory	Anti- IFNγ	emapalumab	NCT04324021
JAK-STAT signaling	Cytokine signaling	JAK1/JAK2 inhibitors	baricitinib	14 studies https://clinicaltrials.gov/ct2/results?cond=COVID-19&term=Baricitinib&cntry=&state=&city=&dist=
			ruxolitinib	20 studies https://clinicaltrials.gov/ct2/results?cond=COVID-19&term=Ruxolitinib&cntry=&state=&city=&dist=&Search=Search
		JAK3 inhibitor	tofacitinib	5 studies https://clinicaltrials.gov/ct2/results?cond=COVID-19&term=Tofacitinib&cntry=&state=&city=&dist=&Search=Search
GM-CSF	Pro-inflammatory	Anti-GM-CSF	otilimab	NCT04376684
CCR2	Monocyte recruitment	Anti-CCR2	cenicriviroc	NCT04500418 NCT04488081
CCR5	Monocyte and T-cell recruitment	Anti-CCR5	leronlimab	NCT04343651; NCT04347239;
Bruton tyrosine kinase (BTK)	B-cell receptor signaling, Toll-like receptors activation	anti-BTK	acalabrutinib	4 studies https://clinicaltrials.gov/ct2/results?cond=covid&term=Acalabrutinib&cntry=&state=&city=&dist=

Clinical trials are indicated by clinicaltrials.gov identifier (NCT) number. For drugs whose clinical trials exceed three, the total number of trials available at the moment of submission is reported, with links leading to the relative trial list at clinicaltrials.gov. IL-6, interleukin-6; IL-1β, interleukin-1 beta; IL-1 βR, IL-1β receptor; CCR, CC- chemokine receptor; IFNγ, interferon gamma; JAK, Janus kinase; STAT, signal transducer and activator of transcription; NETs, neutrophil extracellular traps. N/A, not applicable.

## Discussion

The hypothesis that severe COVID-19 may create a microenvironment favorable to cancer recurrence stems from the authors’ observation that several factors activated during coronavirus infection have been previously implicated in tumorigenesis and metastatic relapse. Recent studies on protein-protein interactions during COVID-19 revealed that common cancer pathways were targeted by SARS-CoV-2, including those involved in cell cycle progression, metabolism and epigenetics (86). However, the interactions between SARS-CoV-2, cancer cells and the immune system are currently unknown and will need to be investigated in detail, possibly with the use of complex *in vitro* models that reproduce multi-cellular microenvironments (87). By contrast, *in vivo* studies to explore COVID-19 and cancer recurrence would likely be challenging, as they should employ mice with multiple genetic modifications predisposing to both COVID-19 and cancer (such as mice transgenic for hACE2 and ErbB2/Neu). In parallel to preclinical studies, in our opinion it will be crucial to investigate all the clinical effects of COVID-19 in cancer patients. While the first large studies in this field have focused on the susceptibility and mortality of COVID-19 in cancer patients (3, 88, 89), new studies are investigating the relationships between anticancer therapies/interventions, single cancer types and immunological status. Additionally, it will be important to assess the long-term effects of severe COVID-19 in patients either with an active cancer, in remission or with a previous history of cancer. Understanding the links between COVID-19 and cancer recurrence may be a challenging task. For example, patients with severe COVID-19 often present concomitant clinical conditions predisposing to cancer recurrence (such as obesity or an immune-compromised state) that may complicate the evaluation of individual risk factors. Nevertheless, a recently launched observational study (CAPTURE, COVID-19 antiviral response in a pan-tumor immune monitoring study) (90) will assess long-term SARS-CoV-2 sequelae on cancer patients including the impact on cancer outcomes, helping to reveal potential effects of COVID-19 on cancer recurrence. In case future studies will confirm a link between severe COVID-19 and tumor recurrence, this finding may be used to schedule personalized treatments and follow-up programs for patients with both conditions. For example, prolonged anti-inflammatory therapies may be evaluated for cancer patients that experienced SARS-CoV-2 infection. Also, the use of drugs with double anticancer/anti-inflammatory action such as acalabrutinib (a Bruton’s tyrosine kinase inhibitor) or leronlimab (an antibody against CCR5 with anti-metastatic activity) is currently being evaluated in the COVID-19 setting (**Table 1**) and in the future may find an increased use the treatment of cancer patients with COVID-19. Altogether, the observations presented in this Perspective suggest a possible link between COVID-19, inflammation and immune-mediated tumor reawakening that, if confirmed by future studies, may have important implications for the treatment and the long-term management of cancer patients.

## Data Availability Statement

The original contributions presented in the study are included in the article/supplementary material. Further inquiries can be directed to the corresponding author.

## Author Contributions

AZ, FF, MLDA, and MBa wrote the manuscript. RR provided essential contribution in editing the manuscript. MBi provided essential expertise. All authors contributed to the article and approved the submitted version.

## Funding

This work was supported by an Italian Association for Cancer Research (AIRC) Investigator Grant to AZ (AIRC IG 2017 Ref: 20744).

## Conflict of Interest

The authors declare that the research was conducted in the absence of any commercial or financial relationships that could be construed as a potential conflict of interest.

## References

[1] Saini KS Tagliamento M Lambertini M McNally R Romano M Leone M Mortality in patients with cancer and COVID-19: A systematic review and pooled analysis of 52 studies. Eur J Cancer (2020) 139:43–50. 10.1016/j.ejca.2020.08.011 32971510 PMC7467090

[2] Archer JE Odeh A Ereidge S Salem HK Jones GP Gardner A Mortality and pulmonary complications in patients undergoing surgery with perioperative SARS-CoV-2 infection: an international cohort study. Lancet (2020) 396:27–38. 10.1016/S0140-6736(20)31182-X 32479829 PMC7259900

[3] Kuderer NM Choueiri TK Shah DP Shyr Y Rubinstein SM Rivera DR Clinical impact of COVID-19 on patients with cancer (CCC19): a cohort study. Lancet (2020) 395:1907–18. 10.1016/S0140-6736(20)31187-9 PMC7255743 32473681

[4] Lee LY Cazier J-B Starkey T Briggs SE Arnold R Bisht V COVID-19 prevalence and mortality in patients with cancer and the effect of primary tumour subtype and patient demographics: a prospective cohort study. Lancet Oncol (2020) 21:1309–16. 10.1016/S1470-2045(20)30442-3 PMC7444972 32853557

[5] Robilotti EV Babady NE Mead PA Rolling T Perez-Johnston R Bernardes M Determinants of COVID-19 disease severity in patients with cancer. Nat Med (2020) 26(8):1218–23. 10.1038/s41591-020-0979-0 PMC7785283 32581323

[6] Seyfried TN Huysentruyt LC On the origin of cancer metastasis. Crit Rev Oncogen (2013) 18(1-2):43. 10.1615/CritRevOncog.v18.i1-2.40 PMC3597235 23237552

[7] Phan TG Croucher PI The dormant cancer cell life cycle. Nat Rev Cancer (2020) 20(7):398–411. 10.1038/s41568-020-0263-0 32488200

[8] Goddard ET Bozic I Riddell SR Ghajar CM Dormant tumour cells, their niches and the influence of immunity. Nat Cell Biol (2018) 20(11):1240–9. 10.1038/s41556-018-0214-0 30361702

[9] Grivennikov SI Greten FR Karin M Immunity, inflammation, and cancer. Cell (2010) 140(6):883–99. 10.1016/j.cell.2010.01.025 PMC2866629 20303878

[10] Quail DF Olson OC Bhardwaj P Walsh LA Akkari L Quick ML Obesity alters the lung myeloid cell landscape to enhance breast cancer metastasis through IL5 and GM-CSF. Nat Cell Biol (2017) 19(8):974–87. 10.1038/ncb3578 PMC6759922 28737771

[11] Hiller JG Perry NJ Poulogiannis G Riedel B Sloan EK Perioperative events influence cancer recurrence risk after surgery. Nat Rev Clin Oncol (2018) 15(4):205. 10.1038/nrclinonc.2017.194 29283170

[12] Yan L Cai Q Xu Y The ubiquitin-CXCR4 axis plays an important role in acute lung infection-enhanced lung tumor metastasis. Clin Cancer Res (2013) 19(17):4706–16. 10.1158/1078-0432.CCR-13-0011 PMC3766445 23690484

[13] Albrengues J Shields MA Ng D Park CG Ambrico A Poindexter ME Neutrophil extracellular traps produced during inflammation awaken dormant cancer cells in mice. Science (2018) 361(6409):eaao4227 10.1126/science.aao4227 30262472 PMC6777850

[14] De Cock JM Shibue T Dongre A Keckesova Z Reinhardt F Weinberg RA Inflammation Triggers Zeb1-Dependent Escape from Tumor Latency. Cancer Res (2016) 76(23):6778–84. 10.1158/0008-5472.CAN-16-0608 PMC5135644 27530323

[15] El Rayes T Catena R Lee S Stawowczyk M Joshi N Fischbach C Lung inflammation promotes metastasis through neutrophil protease-mediated degradation of Tsp-1. Proc Natl Acad Sci USA (2015) 112(52):16000–5. 10.1073/pnas.1507294112 PMC4703007 26668367

[16] Erickson JJ Lu P Wen S Hastings AK Gilchuk P Joyce S Acute Viral Respiratory Infection Rapidly Induces a CD8+ T Cell Exhaustion-like Phenotype. J Immunol (2015) 195(9):4319–30. 10.4049/jimmunol.1403004 PMC4733528 26401005

[17] Hoffmann M Kleine-Weber H Schroeder S Kruger N Herrler T Erichsen S SARS-CoV-2 Cell Entry Depends on ACE2 and TMPRSS2 and Is Blocked by a Clinically Proven Protease Inhibitor. Cell (2020) 181(2):271–80 e8. 10.1016/j.cell.2020.02.052 32142651 PMC7102627

[18] Du L He Y Zhou Y Liu S Zheng B-J Jiang S The spike protein of SARS-CoV—a target for vaccine and therapeutic development. Nat Rev Microbiol (2009) 7(3):226–36. 10.1038/nrmicro2090 PMC2750777 19198616

[19] Rivellese F Prediletto E ACE2 at the centre of COVID-19 from paucisymptomatic infections to severe pneumonia. Autoimmun Rev (2020) 19(6):102536. 10.1016/j.autrev.2020.102536 32251718 PMC7195011

[20] Zhang Q Lu S Li T Yu L Zhang Y Zeng H ACE2 inhibits breast cancer angiogenesis via suppressing the VEGFa/VEGFR2/ERK pathway. J Exp Clin Cancer Res (2019) 38(1):173. 10.1186/s13046-019-1156-5 31023337 PMC6482513

[21] Feng Y Wan H Liu J Zhang R Ma Q Han B The angiotensin-converting enzyme 2 in tumor growth and tumor-associated angiogenesis in non-small cell lung cancer. Oncol Rep (2010) 23(4):941–8. 10.3892/or_00000718 20204277

[22] Yu C Tang W Wang Y Shen Q Wang B Cai C Downregulation of ACE2/Ang-(1-7)/Mas axis promotes breast cancer metastasis by enhancing store-operated calcium entry. Cancer Lett (2016) 376(2):268–77. 10.1016/j.canlet.2016.04.006 27063099

[23] Takimoto-Ohnishi E Murakami K Renin–angiotensin system research: from molecules to the whole body. J Physiol Sci (2019) 69(4):581–7. 10.1007/s12576-019-00679-4 PMC10717639 31028527

[24] Franco R Rivas-Santisteban R Serrano-Marín J Rodríguez-Pérez AI Labandeira-García JL Navarro G SARS-CoV-2 as a Factor to Disbalance the Renin–Angiotensin System: A Suspect in the Case of Exacerbated IL-6 Production. J Immunol (2020) 205(5):1198–206. 10.4049/jimmunol.2000642 32680957

[25] Delpino M Quarleri J SARS-CoV-2 Pathogenesis: Imbalance in the Renin-Angiotensin System Favors Lung Fibrosis. Front Cell Infect Microbiol (2020) 10:340. 10.3389/fcimb.2020.00340 32596170 PMC7303284

[26] Hirano T Murakami M COVID-19: A New Virus, but a Familiar Receptor and Cytokine Release Syndrome. Immunity (2020) 52(5):731–3. 10.1016/j.immuni.2020.04.003 PMC7175868 32325025

[27] Murakami M Kamimura D Hirano T Pleiotropy and Specificity: Insights from the Interleukin 6 Family of Cytokines. Immunity (2019) 50(4):812–31. 10.1016/j.immuni.2019.03.027 30995501

[28] de Wit E van Doremalen N Falzarano D Munster VJ SARS and MERS: recent insights into emerging coronaviruses. Nat Rev Microbiol (2016) 14(8):523–34. 10.1038/nrmicro.2016.81 PMC7097822 27344959

[29] Taniguchi K Karin M NF-kappaB, inflammation, immunity and cancer: coming of age. Nat Rev Immunol (2018) 18(5):309–24. 10.1038/nri.2017.142 29379212

[30] Huang C Wang Y Li X Ren L Zhao J Hu Y Clinical features of patients infected with 2019 novel coronavirus in Wuhan, China. Lancet (2020) 395(10223):497–506. 10.1016/S0140-6736(20)30183-5 31986264 PMC7159299

[31] Lu R Zhao X Li J Niu P Yang B Wu H Genomic characterisation and epidemiology of 2019 novel coronavirus: implications for virus origins and receptor binding. Lancet (2020) 395(10224):565–74. 10.1016/S0140-6736(20)30251-8 PMC7159086 32007145

[32] Liu J Li S Liu J Liang B Wang X Wang H Longitudinal characteristics of lymphocyte responses and cytokine profiles in the peripheral blood of SARS-CoV-2 infected patients. EBioMedicine (2020) 55:102763. 10.1016/j.ebiom.2020.102763 32361250 PMC7165294

[33] Zhou F Yu T Du R Fan G Liu Y Liu Z Clinical course and risk factors for mortality of adult inpatients with COVID-19 in Wuhan, China: a retrospective cohort study. Lancet (2020) 395(10229):1054–62. 10.1016/S0140-6736(20)30566-3 PMC7270627 32171076

[34] Kang S Tanaka T Narazaki M Kishimoto T Targeting Interleukin-6 Signaling in Clinic. Immunity (2019) 50(4):1007–23. 10.1016/j.immuni.2019.03.026 30995492

[35] Atsumi T Singh R Sabharwal L Bando H Meng J Arima Y Inflammation amplifier, a new paradigm in cancer biology. Cancer Res (2014) 74(1):8–14. 10.1158/0008-5472.CAN-13-2322 24362915

[36] Lee JW Stone ML Porrett PM Thomas SK Komar CA Li JH Hepatocytes direct the formation of a pro-metastatic niche in the liver. Nature (2019) 567(7747):249–52. 10.1038/s41586-019-1004-y PMC6430113 30842658

[37] Li S Wang N Brodt P Metastatic cells can escape the proapoptotic effects of TNF-alpha through increased autocrine IL-6/STAT3 signaling. Cancer Res (2012) 72(4):865–75. 10.1158/0008-5472.CAN-11-1357 22194466

[38] Chan LC Li CW Xia W Hsu JM Lee HH Cha JH IL-6/JAK1 pathway drives PD-L1 Y112 phosphorylation to promote cancer immune evasion. J Clin Invest (2019) 129(8):3324–38. 10.1172/JCI126022 PMC6668668 31305264

[39] Sullivan NJ Sasser AK Axel AE Vesuna F Raman V Ramirez N Interleukin-6 induces an epithelial-mesenchymal transition phenotype in human breast cancer cells. Oncogene (2009) 28(33):2940–7. 10.1038/onc.2009.180 PMC5576031 19581928

[40] Huang L Hu B Ni J Wu J Jiang W Chen C Transcriptional repression of SOCS3 mediated by IL-6/STAT3 signaling via DNMT1 promotes pancreatic cancer growth and metastasis. J Exp Clin Cancer Res (2016) 35:27. 10.1186/s13046-016-0301-7 26847351 PMC4743194

[41] Itoh H Kadomatsu T Tanoue H Yugami M Miyata K Endo M TET2-dependent IL-6 induction mediated by the tumor microenvironment promotes tumor metastasis in osteosarcoma. Oncogene (2018) 37(22):2903–20. 10.1038/s41388-018-0160-0 29515232

[42] Wang YC Wu YS Hung CY Wang SA Young MJ Hsu TI USP24 induces IL-6 in tumor-associated microenvironment by stabilizing p300 and beta-TrCP and promotes cancer malignancy. Nat Commun (2018) 9(1):3996. 10.1038/s41467-018-06178-1 30266897 PMC6162259

[43] Balamurugan K Mendoza-Villanueva D Sharan S Summers GH Dobrolecki LE Lewis MT C/EBPdelta links IL-6 and HIF-1 signaling to promote breast cancer stem cell-associated phenotypes. Oncogene (2019) 38(20):3765–80. 10.1038/s41388-018-0516-5 PMC6437025 30262865

[44] Gallo M Frezzetti D Roma C Chicchinelli N Barbieri A Arra C RANTES and IL-6 cooperate in inducing a more aggressive phenotype in breast cancer cells. Oncotarget (2018) 9(25):17543–53. 10.18632/oncotarget.24784 PMC5915136 29707128

[45] Rodrigues CFD Serrano E Patricio MI Val MM Albuquerque P Fonseca J Stroma-derived IL-6, G-CSF and Activin-A mediated dedifferentiation of lung carcinoma cells into cancer stem cells. Sci Rep (2018) 8(1):11573. 10.1038/s41598-018-29947-w 30069023 PMC6070555

[46] Wang T Song P Zhong T Wang X Xiang X Liu Q The inflammatory cytokine IL-6 induces FRA1 deacetylation promoting colorectal cancer stem-like properties. Oncogene (2019) 38(25):4932–47. 10.1038/s41388-019-0763-0 PMC6756002 30804456

[47] Wang Y Zong X Mitra S Mitra AK Matei D Nephew KP IL-6 mediates platinum-induced enrichment of ovarian cancer stem cells. JCI Insight (2018) 3(23):e122360. 10.1172/jci.insight.122360 PMC6328027 30518684

[48] Gross AC Cam H Phelps DA Saraf AJ Bid HK Cam M IL-6 and CXCL8 mediate osteosarcoma-lung interactions critical to metastasis. JCI Insight (2018) 3(16):e99791. 10.1172/jci.insight.99791 PMC6141177 30135299

[49] Meyer F Samson É Douville P Duchesne T Liu G Bairati I Serum prognostic markers in head and neck cancer. Clin Cancer Res (2010) 16(3):1008–15. 10.1158/1078-0432.CCR-09-2014 20103685

[50] Semesiuk N Zhylchuk A Bezdenezhnykh N Lykhova A Vorontsova A Zhylchuk V Disseminated tumor cells and enhanced level of some cytokines in bone marrow and peripheral blood of breast cancer patients as predictive factors of tumor progression. Exp Oncol (2013) 35,№ 4):295–302. 24382441

[51] Chang T-S Wu Y-C Chi C-C Su W-C Chang P-J Lee K-F Activation of IL6/IGFIR confers poor prognosis of HBV-related hepatocellular carcinoma through induction of OCT4/NANOG expression. Clin Cancer Res (2015) 21(1):201–10. 10.1158/1078-0432.CCR-13-3274 25564572

[52] Finkel KA Warner KA Kerk S Bradford CR McLean SA Prince ME IL-6 inhibition with MEDI5117 decreases the fraction of head and neck cancer stem cells and prevents tumor recurrence. Neoplasia (2016) 18(5):273–81. 10.1016/j.neo.2016.03.004 PMC4887598 27237319

[53] Lai S-C Su Y-T Chi C-C Kuo Y-C Lee K-F Wu Y-C DNMT3b/OCT4 expression confers sorafenib resistance and poor prognosis of hepatocellular carcinoma through IL-6/STAT3 regulation. J Exp Clin Cancer Res (2019) 38(1):1–18. 10.1186/s13046-019-1442-2 31771617 PMC6878666

[54] Liao D Liu Z Wrasidlo WJ Luo Y Nguyen G Chen T Targeted therapeutic remodeling of the tumor microenvironment improves an HER-2 DNA vaccine and prevents recurrence in a murine breast cancer model. Cancer Res (2011) 71(17):5688–96. 10.1158/0008-5472.CAN-11-1264 21784871

[55] Jones SA Jenkins BJ Recent insights into targeting the IL-6 cytokine family in inflammatory diseases and cancer. Nat Rev Immunol (2018) 18(12):773–89. 10.1038/s41577-018-0066-7 30254251

[56] Fisher DT Appenheimer MM Evans SS The two faces of IL-6 in the tumor microenvironment. Semin Immunol 26(1):38–47. 10.1016/j.smim.2014.01.008 PMC3970580 24602448

[57] Cavalli G De Luca G Campochiaro C Della-Torre E Ripa M Canetti D Interleukin-1 blockade with high-dose anakinra in patients with COVID-19, acute respiratory distress syndrome, and hyperinflammation: a retrospective cohort study. Lancet Rheumatol (2020) 2:E325–31. 10.1016/S2665-9913(20)30127-2 32501454 PMC7252085

[58] Mantovani A Barajon I Garlanda C IL-1 and IL-1 regulatory pathways in cancer progression and therapy. Immunol Rev (2018) 281(1):57–61. 10.1111/imr.12614 29247996 PMC5922413

[59] Bent R Moll L Grabbe S Bros M Interleukin-1 beta—a friend or foe in malignancies? Int J Mol Sci (2018) 19(8):2155. 10.3390/ijms19082155 PMC6121377 30042333

[60] Wellenstein MD Coffelt SB Duits DEM van Miltenburg MH Slagter M de Rink I Loss of p53 triggers WNT-dependent systemic inflammation to drive breast cancer metastasis. Nature (2019) 572(7770):538–42. 10.1038/s41586-019-1450-6 PMC6707815 31367040

[61] Leach J Morton JP Sansom OJ Neutrophils: Homing in on the myeloid mechanisms of metastasis. Mol Immunol (2019) 110:69–76. 10.1016/j.molimm.2017.12.013 29269005 PMC6544568

[62] Rapoport BL Steel HC Theron AJ Smit T Anderson R Role of the Neutrophil in the Pathogenesis of Advanced Cancer and Impaired Responsiveness to Therapy. Molecules (2020) 25(7):1618. 10.3390/molecules25071618 PMC7180559 32244751

[63] Lu C Redd PS Lee JR Savage N Liu K The expression profiles and regulation of PD-L1 in tumor-induced myeloid-derived suppressor cells. Oncoimmunology (2016) 5(12):e1247135. 10.1080/2162402X.2016.1247135 28123883 PMC5214087

[64] Zhu J Powis de Tenbossche CG Cane S Colau D van Baren N Lurquin C Resistance to cancer immunotherapy mediated by apoptosis of tumor-infiltrating lymphocytes. Nat Commun (2017) 8(1):1404. 10.1038/s41467-017-00784-1 29123081 PMC5680273

[65] mkBarnes BJ Adrover JM Baxter-Stoltzfus A Borczuk A Cools-Lartigue J Crawford JM Targeting potential drivers of COVID-19: Neutrophil extracellular traps. J Exp Med (2020) 217(6):e20200652. 10.1084/jem.20200652 32302401 PMC7161085

[66] Zuo Y Yalavarthi S Shi H Gockman K Zuo M Madison JA Neutrophil extracellular traps in COVID-19. JCI Insight (2020) 5:e138999. 10.1172/jci.insight.138999 PMC7308057 32329756

[67] Middleton EA He XY Denorme F Campbell RA Ng D Salvatore SP Neutrophil Extracellular Traps (NETs) Contribute to Immunothrombosis in COVID-19 Acute Respiratory Distress Syndrome. Blood (2020) 136:1169–79. 10.1182/blood.2020007008 PMC7472714 32597954

[68] Radermecker C Detrembleur N Guiot J Cavalier E Henket M d’Emal C Neutrophil extracellular traps infiltrate the lung airway, interstitial, and vascular compartments in severe COVID-19. J Exp Med (2020) 217(12):e20201012. 10.1084/jem.20201012 32926097 PMC7488867

[69] Veras FP Pontelli M Silva C Toller-Kawahisa J de Lima M Nascimento D SARS-CoV-2 triggered neutrophil extracellular traps (NETs) mediate COVID-19 pathology. J Exp Med (2020) 217:Pe20201129. 10.1101/2020.06.08.20125823 PMC7488868 32926098

[70] Teijeira A Garasa S Gato M Alfaro C Migueliz I Cirella A CXCR1 and CXCR2 Chemokine Receptor Agonists Produced by Tumors Induce Neutrophil Extracellular Traps that Interfere with Immune Cytotoxicity. Immunity (2020) 52:856–71. 10.1016/j.immuni.2020.03.001 32289253

[71] Cools-Lartigue J Spicer J McDonald B Gowing S Chow S Giannias B Neutrophil extracellular traps sequester circulating tumor cells and promote metastasis. J Clin Invest (2013) 123:3446–58. 10.1172/JCI67484 PMC3726160 23863628

[72] Takesue S Ohuchida K Shinkawa T Otsubo Y Matsumoto S Sagara A Neutrophil extracellular traps promote liver micrometastasis in pancreatic ductal adenocarcinoma via the activation of cancerassociated fibroblasts. Int J Oncol (2020) 56(2):596–605. 10.3892/ijo.2019.4951 31894273

[73] Vabret N Britton GJ Gruber C Hegde S Kim J Kuksin M Immunology of COVID-19: Current State of the Science. Immunity (2020) 52(6):910–41. 10.1016/j.immuni.2020.05.002 PMC7200337 32505227

[74] Chen Z Wherry EJ T cell responses in patients with COVID-19. Nat Rev Immunol (2020) 20:529–36. 10.1038/s41577-020-0402-6 PMC7389156 32728222

[75] Eyles J Puaux AL Wang X Toh B Prakash C Hong M Tumor cells disseminate early, but immunosurveillance limits metastatic outgrowth, in a mouse model of melanoma. J Clin Invest (2010) 120(6):2030–9. 10.1172/JCI42002 PMC2877955 20501944

[76] Romero I Garrido C Algarra I Collado A Garrido F Garcia-Lora AM T lymphocytes restrain spontaneous metastases in permanent dormancy. Cancer Res (2014) 74(7):1958–68. 10.1158/0008-5472.CAN-13-2084 24531750

[77] Malladi S Macalinao DG Jin X He L Basnet H Zou Y Metastatic Latency and Immune Evasion through Autocrine Inhibition of WNT. Cell (2016) 165(1):45–60. 10.1016/j.cell.2016.02.025 27015306 PMC4808520

[78] Group RC Dexamethasone in hospitalized patients with Covid-19—preliminary report. New Engl J Med (2020) NEJMoa2021436:1–11. 10.1056/NEJMoa2021436

[79] Merad M Martin JC Pathological inflammation in patients with COVID-19: a key role for monocytes and macrophages. Nat Rev Immunol (2020) 20:355–62. 10.1038/s41577-020-0331-4 PMC7201395 32376901

[80] Yeleswaram S Smith P Burn T Covington M Juvekar A Li Y Inhibition of cytokine signaling by ruxolitinib and implications for COVID-19 treatment. Clin Immunol (2020) 218:108517. 10.1016/j.clim.2020.108517 32585295 PMC7308779

[81] Stebbing J Krishnan V de Bono S Ottaviani S Casalini G Richardson PJ Mechanism of baricitinib supports artificial intelligence-predicted testing in COVID-19 patients. EMBO Mol Med (2020) 12:e12697. 10.21203/rs.3.rs-23195/v1 32473600 PMC7300657

[82] Thibaud S Tremblay D Bhalla S Zimmerman B Sigel K Gabrilove J Protective role of Bruton tyrosine kinase inhibitors in patients with chronic lymphocytic leukaemia and COVID-19. Br J Haematol (2020) 190:e73–6. 10.1111/bjh.16863 32433778 PMC7276870

[83] Ghajar CM Metastasis prevention by targeting the dormant niche. Nat Rev Cancer (2015) 15(4):238–47. 10.1038/nrc3910 PMC4842412 25801619

[84] Obradović MM Hamelin B Manevski N Couto JP Sethi A Coissieux M-M Glucocorticoids promote breast cancer metastasis. Nature (2019) 567(7749):540–4. 10.1038/s41586-019-1019-4 30867597

[85] Ungprasert P Srivali N Wijarnpreecha K Charoenpong P Knight EL Non-steroidal anti-inflammatory drugs and risk of venous thromboembolism: a systematic review and meta-analysis. Rheumatology (2015) 54(4):736–42. 10.1093/rheumatology/keu408 25252703

[86] Tutuncuoglu B Cakir M Batra J Bouhaddou M Eckhardt M Gordon DE The Landscape of Human Cancer Proteins Targeted by SARS-CoV-2. Cancer Discovery (2020) 10:916–21. 10.1158/2159-8290.CD-20-0559 PMC7357668 32444466

[87] Fiorini E Veghini L Corbo V Modeling Cell Communication in Cancer With Organoids: Making the Complex Simple. Front Cell Dev Biol (2020) 8:166. 10.3389/fcell.2020.00166 32258040 PMC7094029

[88] Garassino MC Whisenant JG Huang L-C Trama A Torri V Agustoni F COVID-19 in patients with thoracic malignancies (TERAVOLT): first results of an international, registry-based, cohort study. Lancet Oncol (2020) 21:914–22. 10.1016/S1470-2045(20)30314-4 PMC7292610 32539942

[89] Group CC Mortality and pulmonary complications in patients undergoing surgery with perioperative SARS-CoV-2 infection: an international cohort study. Lancet (2020) 396:P27–38. 10.1016/S0140-6736(20)31182-X PMC7259900 32479829

[90] Au L Boos LA Swerdlow A Byrne F Shepherd ST Fendler A Cancer, COVID-19, and antiviral immunity: the CAPTURE study. Cell (2020) 183:P4–10. 10.1016/j.cell.2020.09.005 PMC7470737 32979319

